# Alpelisib-Induced Diabetic Ketoacidosis

**DOI:** 10.7759/cureus.14796

**Published:** 2021-05-01

**Authors:** Paul Nguyen, Aya Musa, Julie Samantray

**Affiliations:** 1 Internal Medicine, Wayne State University School of Medicine, Detroit, USA; 2 Endocrinology, Diabetes and Metabolism, Wayne State University School of Medicine, Detroit, USA; 3 Endocrine Oncology, Karmanos Cancer Center, Detroit, USA

**Keywords:** alpelisib, piqray, diabetic ketoacidosis, hyperglycemia, breast cancer, oncology, intensive care, adverse drug events

## Abstract

We present the third case of alpelisib-induced diabetic ketoacidosis. Alpelisib is an antineoplastic agent that inhibits phosphatidylinositol 3-kinase (PI3K), which plays a key role in multiple biological processes such as cell differentiation, proliferation, and survival. Thereby, the inhibition of this pathway should cause antitumor activity. Alpelisib was recently approved by the Food and Drug Administration (FDA) for use in PIK3CA-mutated breast cancer. This mutation is a common indicator of poor prognosis and is also the most commonly mutated gene in hormone receptor (HR)-positive and human epidermal growth factor receptor 2 (HER2)-negative advanced breast cancer. During its trial, ketoacidosis was reported in only 0.7% of patients, with the more common side effects (>20%) being diarrhea (58%), rash (52%), nausea (45%), fatigue (42%), decreased appetite (36%), stomatitis (30%), vomiting (27%), weight loss (27%), and alopecia (20%). As breast cancer is the second most common cancer in women and approximately 40% of HR+/HER2- advanced breast cancer patients have a PIK3CA mutation, alpelisib will be prescribed more by oncologists and, therefore, appropriate screening with fasting plasma glucose, hemoglobin A1c (HbA1C), and monitoring during drug administration is of utmost importance.

## Introduction

Diabetic ketoacidosis is a life-threatening medical condition that affects approximately 30 million people in the United States [1]. On the other hand, breast cancer is the second most common cancer in women and is estimated to have 276,480 new diagnoses with 42,170 deaths in 2020 [2]. Alpelisib (PIQRAY®, Novartis Pharmaceuticals Corporation, Switzerland) is an oral phosphatidylinositol 3-kinase (PI3K) alpha-specific inhibitor that was approved by the Food and Drug Administration (FDA) on May 24, 2019. With its recent approval, alpelisib, in combination with fulvestrant, is a new regimen for patients with hormone receptor (HR)-positive, human epidermal growth factor receptor 2 (HER2)-negative, and PIK3CA-mutated breast cancer.

## Case presentation

We report a rare case of alpelisib-induced diabetic ketoacidosis (DKA) in a 73-year-old white female with no prior personal history or family history of diabetes. She has a history of metastatic breast ductal carcinoma (estrogen-sensitive; human epidermal growth factor receptor 2 (HER2)-negative, and PIK3CA mutated) with lung, liver, and bony involvement. Fulvestrant and palbociclib were the initial treatment, but with disease progression treatment transitioned to capecitabine and subsequently alpelisib at 300 mg daily plus fulvestrant. Four days after starting alpelisib, she developed asthenia, polyuria, polydipsia, nausea, and vomiting. After three additional days, she had a fasting plasma glucose (FPG) of 404 mg/dL and a hemoglobin A1C (HbA1C) of 8.1%. Four days following that, she came back to the clinic with a diffuse maculopapular rash and was found to have an FPG of 707 mg/dL, bicarbonate 13, anion gap of 22 mMol/L, beta-hydroxybutyrate 58.3, and HbA1c of 9.1%. She was then admitted to the medical intensive care unit for the management of acute hyperglycemia and ketoacidosis.

**Figure 1:**
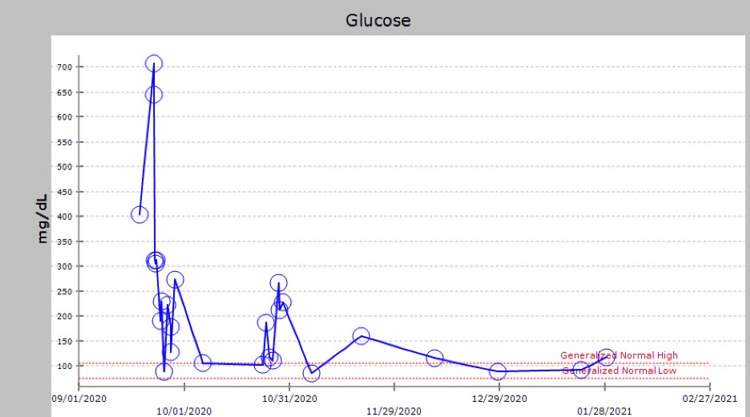
Graph depicting capillary blood glucose (CBG) from the start of alpelisib initiation and throughout the hospital stay and beyond

She was treated with intravenous insulin infusion (84 units total daily dose) and then transitioned to subcutaneous insulin dosing within 24 hours. On Day 2 of admission, the patient had results of C-peptide 1.9 ng/mL and glucose 230 mg/dL. Her insulin requirement decreased over time. The diffuse maculopapular rash persisted despite regular use of topical hydrocortisone, and the patient was started on oral prednisone on Day 5 for a total of eight days with a tapering dose and the complete resolution of the rash. Pancreatic islet cell antibodies were negative and other antibodies were not available. Alpelisib was discontinued and her glucose profile normalized, and she came off of insulin. She was discharged home after six days on metformin 1000 mg twice daily. On subsequent visits with her oncologist, she began a regimen with vinorelbine, which she has tolerated well. Her sugar has since been in the non-diabetic range (Figure 1).

## Discussion

Alpelisib (PIQRAY) is an oral phosphatidylinositol 3 kinase (PI3K) alpha-specific inhibitor and, in combination with fulvestrant, has synergistic antitumor activity. The altered PI3K pathway occurs in approximately 58% of breast tumors and 44% of all tumors, making it an important target for drug therapy [3]. PI3K plays a central role in a cell’s physiology. It mediates growth factor signaling during growth and critical cellular processes, including glucose homeostasis, lipid metabolism, protein synthesis, and cell proliferation and survival [4-5].

The PI3K/Akt signaling pathway is key to glucose homeostasis in many ways. First, the PI3K/Akt pathway in insulin-mediated glucose uptake: binding of insulin to its receptor leads to the autophosphorylation of the receptor and subsequently the phosphorylation of insulin receptor substrate proteins on its tyrosine residues. This initiates the PI3K/Akt pathway. The activated PI3K, through a cascade of subsequent steps, triggers the phosphorylation of Akt. The activated Akt, which is primarily expressed in insulin-responsive tissues (skeletal muscle, adipose tissues, and liver), promotes the translocation of glucose transporter 4 (GLUT4) [6] to the cellular membrane resulting in glucose inflow. Additionally, the action on glucose metabolism: Akt influences downstream substrates such as Forkhead box protein O1 (FoxO1) transcription factor and glycogen synthase kinase 3(GSK3). FoxO1 increases gluconeogenesis. Akt inhibits FoxO1 and thus reduces glucose levels. Akt increases glycogen synthesis in liver and skeletal muscle through the inhibition of glycogen synthase kinase 3. Akt regulates glycolysis by stimulating hexokinase, which converts glucose to glucose 6-phosphate. The PI3K/Akt signaling pathway regulates glucose metabolism through FoxO1 and GSK3 [5]. Lastly, altered Akt activity in pancreatic β cells has been shown to cause a decrease in beta-cell mass and function resulting in insulin secretory dysfunction. This is similar to the insulin resistance and impaired glucose tolerance seen in the development of type 2 diabetes mellitus (T2DM). The PI3K/Akt pathway is altered in obesity and T2DM, reducing insulin secretion and β cell function [7]. Smaller tumor volumes may be achieved by reducing insulin levels, thereby decreasing insulin’s ability to activate insulin receptors in tumors [8].

The FDA approved alpelisib (PIQRAY), in combination with fulvestrant, for patients with hormone receptor (HR)-positive, HER2-negative, PIK3CA-mutated breast cancer on May 24, 2019. The phase 3 SOLAR-1 trial (Study Assessing the Efficacy and Safety of Alpelisib Plus Fulvestrant in Men and Postmenopausal Women With Advanced Breast Cancer Which Progressed on or After Aromatase Inhibitor Treatment) randomized 572 patients. This study enrolled patients with controlled type 2 diabetes only, which was defined as FPG ≤140 mg/dL and HbA1c ≤6.4%. Grade 3 or 4 hyperglycemia was the most common adverse effect. In cases of FPG above 500 mg/dL (grade 4 hyperglycemia), the experimental drug was discontinued altogether. Sixty-three percent of patients started on alpelisib develop hyperglycemia and 14% develop a rash. However, grade 4 hyperglycemia was reported in only 3.9 % and DKA in 0.7% [9].

## Conclusions

Given that the PIK3CA mutation in breast cancer is fairly common, it is anticipated that there will be increased use of PI3K inhibitors such as alpelisib. Alpelisib can cause new-onset diabetes and/or exacerbate pre-existing diabetes. Hyperglycemia from this agent is fortunately reversible with the discontinuation of the drug. Early recognition of the symptoms, monitoring glucose, and multidisciplinary care is key to the successful management of the life-threatening condition of DKA.
